# Salt Stress Enhanced Bioactivity of Quinoa Leaf Extracts: An In Vitro and In Silico Study of Acetylcholinesterase and Tyrosinase Inhibition for Sustainable Drug Development

**DOI:** 10.3390/ph18010077

**Published:** 2025-01-10

**Authors:** Narmine Slimani, Soumaya Arraouadi, Hafedh Hajlaoui, Antonio Cid-Samamed, Mohamed Ali Borgi, Mejdi Snoussi

**Affiliations:** 1 Laboratory of Biotechnology and Biomonitoring of the Environment and Oasis Ecosystems (LBBEEO), Faculty of Sciences of Gafsa, University of Gafsa, Zarroug, Gafsa 2112, Tunisia; narmine.slimani96@gmail.com (N.S.); borgima@fsgf.ugaf.tn (M.A.B.); 2 Regional Center of Agricultural Research (CRRA) Sidi Bouzid, Gafsa Road Km 5, PB 357, Sidi Bouzid 9100, Tunisia; 3 Laboratory of Valorization of Unconventional Waters, INRGREF, University of Carthage, Road Hedi El Karray, El Menzah IV, PB 10, Ariana 2080, Tunisia; 4 Faculty of Sciences and Technology of Sidi Bouzid, University of Kairouan, Campus University Agricultural City, Sidi Bouzid 9100, Tunisia; bio.hafedh@gmail.com; 5 Laboratory of Plant-Soil-Environment Interactions, LR21ES01, Faculty of Sciences of Tunis, University of Tunis EL Manar, Tunis 2092, Tunisia; 6 Departamento de Química Física, Facultade de Ciencias, Universidade de Vigo, Campus de As Lagoas s/n, 32004 Ourense, Spain; 7 Department of Biology, College of Science, Hail University, P.O. Box 2440, Ha’il 2440, Saudi Arabia; snmejdi@yahoo.fr; 8 Laboratory of Genetics, Biodiversity and Valorization of Bio-Resources (LR11ES41), Higher Institute of Biotechnology of Monastir, University of Monastir, Avenue Tahar Haddad, BP74, Monastir 5000, Tunisia

**Keywords:** quinoa, salt stress, acetylcholinesterase activity, tyrosinase activity, in vitro study, in silico study

## Abstract

**Background:** Quinoa is recognized for its nutritional and pharmacological properties. This study aims to investigate the impact of salt stress induced by varying concentrations of sodium chloride (NaCl) on the production of phenolic compounds and their biological activities in different quinoa accessions. **Method:** Leaves from three quinoa accessions (Q4, Q24, and Q45) cultivated under increasing NaCl treatments were subjected to chemical analysis using ethanol and water extract. The concentrations of various phenolic compounds, including polyphenols, tannins, anthocyanins, and flavonoids, were quantified. HPLC-DAD-ESI-MS/MS was employed to identify the major compounds in the water extract. Additionally, antioxidants (ABTS and FRAP), anti-tyrosinase, and anti-acetylcholinesterase effects were assessed using in vitro and in silico approaches. **Results:** NaCl treatment significantly increased the levels of phenolic compounds across all quinoa accessions. The Q45 accession exhibited the highest accumulation of these compounds, particularly in the aqueous extracts at the 200 mM NaCl concentration. Increases were observed in flavonoids (144%), anthocyanins (125%), tannins (89%), and total polyphenols (65%) relative to controls. HPLC-DAD-ESI-MS/MS analysis corroborated these findings, showing that the main compounds also increased with higher NaCl concentrations. Furthermore, the biological efficacy tests revealed that the IC_50_ values for both tyrosinase and acetylcholinesterase activities decreased with greater salt stress, indicating enhanced enzyme inhibition. The antioxidant activity of these extracts also showed a significant increase as the salt stress levels rose. **Conclusions:** Salt stress not only promotes the production of bioactive phenolic compounds in quinoa leaves but also enhances their inhibitory effects on key enzymes associated with neurodegenerative and pigmentary disorders. These findings suggest that quinoa may serve as a valuable resource for therapeutic applications, particularly under increased salinity conditions.

## 1. Introduction

The rise in synthetic drugs has undeniably revolutionized medicine, providing effective solutions to many diseases. However, this progress comes with significant challenges. The serious side effects, antibiotic resistance, and environmental concerns associated with the production and disposal of these compounds pose critical questions for public health and the sustainability of our planet [1].

Faced with these challenges, the idea of exploring biological molecules, particularly those of plant origin, is emerging as a promising alternative. Rather than attempting to design synthetic molecules with fewer undesirable effects, it is often easier and safer to exploit the therapeutic properties of natural compounds already existing in nature. These biological molecules offer a variety of compounds with different medicinal properties, often comparable or even superior to those of synthetic drugs, with fewer unwanted side effects. This exploration of plant biological molecules represents, therefore, a major opportunity to rethink our medical approach by placing the richness and diversity of nature at the heart of our treatments.

In this context, quinoa (*Chenopodium quinoa* Willd.), a member of the Amaranthaceae family, a plant with multiple virtues, is garnering growing interest for its diverse parts: seeds, leaves, stems, and roots, which are rich in bioactive compounds with potential medicinal properties [2,3]. These parts are abundant in secondary metabolites, including flavonoids, saponins, and phenolic acids [4,5]. These bioactive compounds endow quinoa with various biological activities, including antioxidant, anti-inflammatory, anticancer, and antimicrobial effects [6,7]. In recent years, numerous studies have also highlighted the inhibitory effect of these compounds on tyrosinase and acetylcholinesterase, proving their role in both skin and neurological health. Tyrosinase, an enzyme involved in melanin biosynthesis, plays a critical role in conditions like hyperpigmentation and melasma, where its inhibition by natural compounds, particularly polyphenols and flavonoids, has shown therapeutic potential. Flavonoids, with their antioxidant properties, are particularly effective in reducing tyrosinase activity, thereby controlling excess pigmentation [8]. On the other hand, acetylcholinesterase, an enzyme responsible for the breakdown of acetylcholine in the nervous system, is associated with neurodegenerative diseases such as Alzheimer’s. Polyphenols have been found to inhibit acetylcholinesterase activity, enhancing acetylcholine levels and potentially offering a protective effect against cognitive decline [9].

This plant also has exceptional nutritional properties, particularly in its seeds, which are the most commonly consumed part of the plant. The seeds are rich in high-quality protein, containing all essential amino acids, making them a valuable source of plant-based protein [10]. They are also abundant in dietary fiber, which supports digestive health, and contain important minerals like magnesium, iron, and zinc, which contribute to bone health, oxygen transport, and immune function [6]. In addition to the seeds, quinoa leaves are also edible and highly nutritious. The leaves are a good source of vitamins (such as vitamins C and A), minerals, and antioxidants, which help combat oxidative stress and promote overall health [2,3]. This versatility makes quinoa an excellent candidate for addressing nutritional deficiencies and enhancing human health.

However, in their natural habitat, quinoa plants are exposed to a wide range of environmental factors, which are further exacerbated by climate change [11]. Among these factors, soil salinity stands out as a major stress that can significantly affect plant growth and survival by reducing the efficiency of photosynthetic processes and disrupting their overall metabolism, thereby inducing oxidative stress [12,13]. Soil salinity also disrupts the ionic and osmotic balance and reduces the availability of essential nutrients, making their uptake more difficult [14,15,16].

At the metabolic level, salinity can induce notable changes in the biosynthesis of bioactive compounds such as polyphenols, flavonoids, and other compounds. Indeed, many studies have shown that the production of these compounds can either increase or decrease depending on the stress intensity [17,18]. Salt stress can also profoundly affect the quality of metabolites produced by plants, affecting their efficacy in various applications. This stress can modify the chemical structure of metabolites, changing their concentration and affecting their pharmacological properties [19]. In this context, it is imperative to understand the specific effects of salt stress on the production and efficacy of biological molecules extracted from quinoa leaves. Therefore, this study aims to quantify the total concentrations of polyphenols, flavonoids, and tannins and identify the major compounds through HPLC-DAD-ESI-MS/MS analysis. Additionally, this study will evaluate the salt resistance of these molecules, focusing, in particular, on their antioxidant activity using two tests: FRAP (Ferric Reducing Antioxidant Power) and ABTS (2,2′-Azinobis(3-ethylbenzothiazoline-6-sulfonic acid)) and their inhibitory properties on the enzymes tyrosinase and acetylcholinesterase, which play a key role in the treatment of pigmentary and neurodegenerative disorders, respectively. These analyses offer promising prospects for the continued reliability of the use of plant compounds in the therapeutic field, even under restrictive environmental conditions.

## 2. Results

### 2.1. Salt Stress Effect on Phenolic Compound Contents

The effects analysis of salt stress on the metabolite content in quinoa accessions revealed a consistent increase in polyphenols, flavonoids, and tannins as NaCl concentration increased from 50 mM to 200 mM, as demonstrated in Table 1. In addition, water extraction yielded higher metabolite levels than ethanol, indicating the predominantly polar nature of these compounds. The Q45 accession showed the highest metabolite accumulation, especially for the aqueous extract, possibly indicating a superior salt tolerance. Under 200 mM NaCl, the contents were stimulated by 144% for flavonoids, 89% for tannins, and 65% for total polyphenols relative to the control. In contrast, the Q4 accession presented the lowest concentrations and stimulation percentages with 15% for flavonoids, 70% for tannins, and 89% for total polyphenols under the same treatment and solvent. These findings highlight the significant effect of salt stress on the production of secondary metabolites in quinoa, with cultivar-specific responses and the importance of the choice of extraction solvent.

### 2.2. HPLC-DAD-ESI-MS/MS Analysis of the Main Compounds of Water Extract

The effect of salt stress on the chemical composition of the studied accessions revealed the presence of five major compounds, as shown in Table 2. These compounds showed a significant stimulation in response to salt stress. For the Q4 accession, Hhdp-galloyl glucose was strongly enhanced, increasing from 1.14% to 9.51%, corresponding to a remarkable 734% increase. Similarly, vanillic acid showed a significant increase from 4.16% to 18.11% (335% increase) and caffeic acid increased from 11.45% to 28.51% (149% increase). In the Q24 accession, *p*-coumaric acid showed a remarkable increase from 4.37% to 19.4% (344% increase). Furthermore, in the Q45 accession, *p*-coumaroyl hexose and Hhdp-galloyl glucose exhibited remarkable increases under 200 mM salinity, increasing by 594% and 924%, respectively.

These results indicate, therefore, that Q45 accession has an exceptional ability to accumulate these specific compounds under high salt stress conditions, suggesting an important metabolic adaptation to cope with these adverse conditions.

### 2.3. Evaluation of Enzyme Activities

#### 2.3.1. In Vitro Study

##### Antioxidant Activity Evaluation: ABTS and FRAP Tests

The antioxidant activity of quinoa extracts was evaluated using the ABTS and FRAP tests, as presented in Table 3. The analysis of these results showed that the antioxidant activities of these extracts were strongly influenced by several factors, including saline treatment, accession, solvents used, and their interactions.

In the ABTS test, the analysis of the IC_50_ values revealed that a higher concentration of NaCl in the irrigation water (200 mM) led to a significant decrease in these values for both extracts, reflecting an increase in their antioxidant activities. Under 200 mM treatment, the IC_50_ values decreased from 7.46 ± 0.56 to 7.1 ± 0.11 mg/mL for Q4, from 7.76 ± 0.28 to 5.1 ± 0.4 mg/mL for Q24, and from 4.83 ± 0.32 to 4.5 ± 0.4 mg/mL for Q45. Similarly, for the aqueous extract, the values decreased from 4.3 ± 0.89 to 3.4 ± 0.11 mg/mL for Q4, from 4.26 ± 0.28 to 3.23 ± 0.23 mg/mL for Q24, and from 3.23 ± 0.42 to 2.16 ± 0.17 mg/mL for Q45. The aqueous extract was more effective than the ethanolic extract, with lower IC_50_ values for all the accessions studied, reinforcing the idea that water promotes a better extraction of antioxidant compounds.

The FRAP test also reflects the same trends. IC_50_ values for the ethanolic extract are lower at 200 mM, reaching 0.86 ± 0.23, 0.77 ± 0.02, and 0.57 ± 0.05 for Q4, Q24, and Q45, respectively, and 0.86 ± 0.23, 0.63 ± 0.02, and 0.28 ± 0.02 for the aqueous extract for the same accessions. Water remains the most effective solvent, extracting the compounds more efficiently and with good antioxidant activity. The accession effect also showed a similar trend, with Q45 maintaining the best performance, followed by Q4 and Q24.

In conclusion, water is a more effective solvent than ethanol in extracting antioxidant compounds in these tests. Increasing the salt stress to 200 mM improves the antioxidant activity of the accessions, and Q45 stands out as the accession with the best antioxidant activity in the two tests studied. Although the values obtained are higher than those of the standard BHT, they are still considered significant.

##### Evaluation of Tyrosinase and Acetylcholinesterase Activities

Table 4 summarizes the results related to the effect of salt stress on the tyrosinase and acetylcholinesterase activities of aqueous and ethanolic quinoa extracts. These activities were strongly influenced by the salt treatment, the accessions, the solvents used, and their interactions.

Regarding the tyrosinase activity involved in melanin biosynthesis, statistical analysis showed an elevated inhibition with increasing NaCl concentration, with the lowest IC_50_ values recorded under 200 mM. Furthermore, the aqueous extract performed better, with IC_50_ values ranging from 1.26 ± 0.14 to 2.2 ± 0.05 mg/mL, compared to the ethanolic extract (from 1.5 ± 0.05 to 4.06 ± 0.37 mg/mL). Similarly, Q45 showed an inhibitory potential greater than that of Q24 and Q4. Despite this significant anti-tyrosinase capacity of leaves, it remains less effective than kojic acid (0.178 ± 0.039 mg/mL).

Regarding acetylcholinesterase, the results revealed a similar trend to that observed for the previous enzyme. Indeed, the IC_50_ values decrease with increasing NaCl concentration. A notable exception was observed for the Q4′ ethanolic extract, which exhibited an increased IC_50_ value at 150 and 200 mM, exceeding that of the control treatment. In fact, under 50 mM concentration, only 0.68 mg/mL of this extract can inhibit 50% of acetylcholinesterase activity, but this value increases under 150 and 200 mM to reach 0.83 and 0.9 mg/mL, respectively. Furthermore, a more pronounced inhibitory potential was observed in Q45 for both types of extracts. In addition, water seems to be more efficient, with IC_50_ values ranging from 0.14 ± 0.002 to 0.22 ± 0.02 mg/mL.

These results highlight that salt stress enhances the efficacy of extracts in inhibiting tyrosinase and acetylcholinesterase. This increase in efficacy highlights the potential of these extracts for applications in the prevention and treatment of pigmentary disorders and neurodegenerative disorders.

#### 2.3.2. In Silico Study

To better understand the relationship between the in vitro biological activities of the tested ligands and their structure and to explore potential mechanisms of action, molecular docking studies were performed on two target enzymes: tyrosinase and acetylcholinesterase. These studies allowed us to determine how the structural features of the ligands influence their ability to modulate or inhibit enzymatic activity.

##### Molecular Docking Study Against ‘Acetylcholinesterase’ (PDB: 4ey7)

The docking results revealed that Hhdp-galloyl glucose was the most bioactive phytocompound, followed by *p*-coumaroyl hexose, as shown in Table 5. These two ligands showed anti-acetylcholinesterase potentials higher than the reference ‘galantamine’.

**Figure 1:**
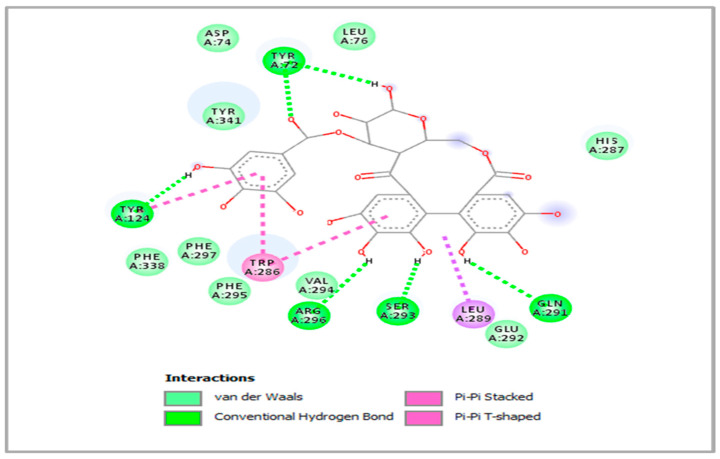
Binding mode of the most active compound Hhdp-galloyl glucose inside the binding cavity of acetylcholinesterase (PBD: 4ey7).

**Figure 2:**
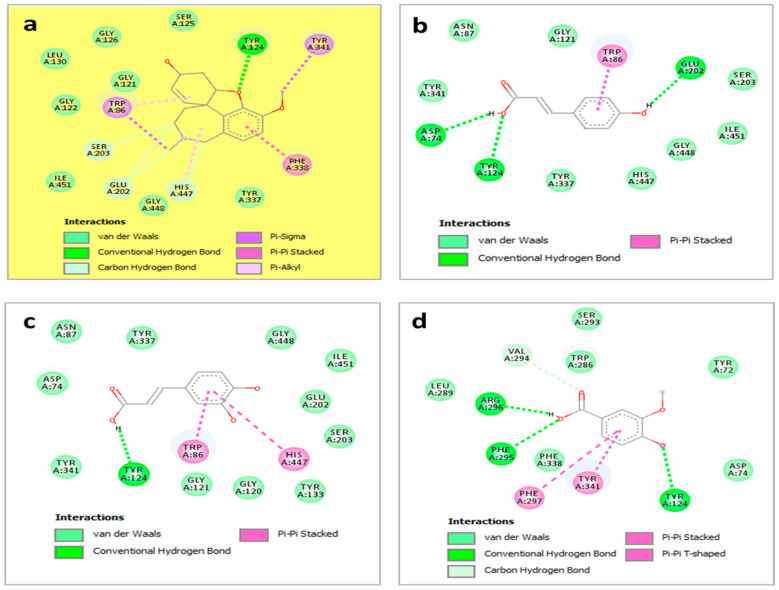

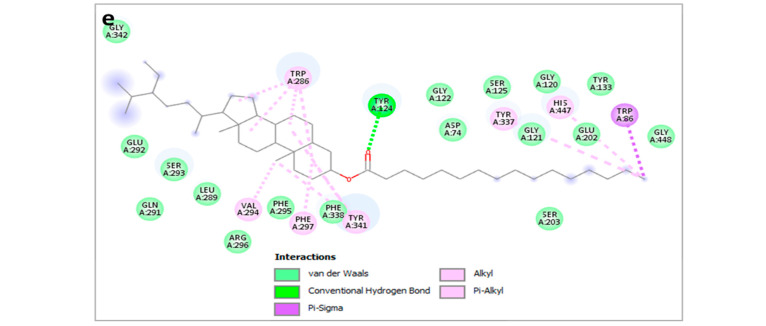
Binding modes of compounds: the reference: Galantamine (**a**), *p*-coumaric acid (**b**), caffeic acid (**c**), vanillic acid (**d**), and *p*-coumaroyl hexose (**e**) within the binding cavity of acetylcholinesterase (PDB: 4ey7).

As can be seen in Figure 1, hhdp-galloyl glucose is involved in six H-bonds with residues Tyr72, Tyr124, Gln291, Ser293, and Arg296 in addition to some other interactions: Pi-Pi stacked with Trp286 and Pi-Pi T-shaped with Tyr124. In contrast, *p*-coumaroyl hexose only formed H-bonds with Tyr124 besides other contacts such as Pi-sigma with Trp86 and Alkyl/Alkyl with the following residue sequence: Trp286, Val294, Phe297, Tyr337, Tyr341, and His447. *p*-coumaric acid, caffeic acid, and vanillic acid were also involved in some significant interactions, especially H-bonds, as illustrated in Figure 2.

##### Molecular Docking Study Against ‘Tyrosinase’ (PDB: 2y9x)

**Figure 3:**
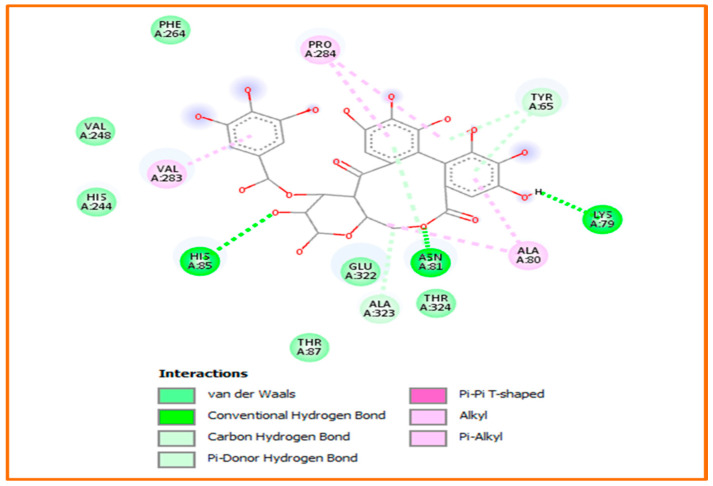
Binding mode of the most active compound Hhdp-galloyl glucose within the tyrosinase cavity (PDB: 2y9x).

**Figure 4:**
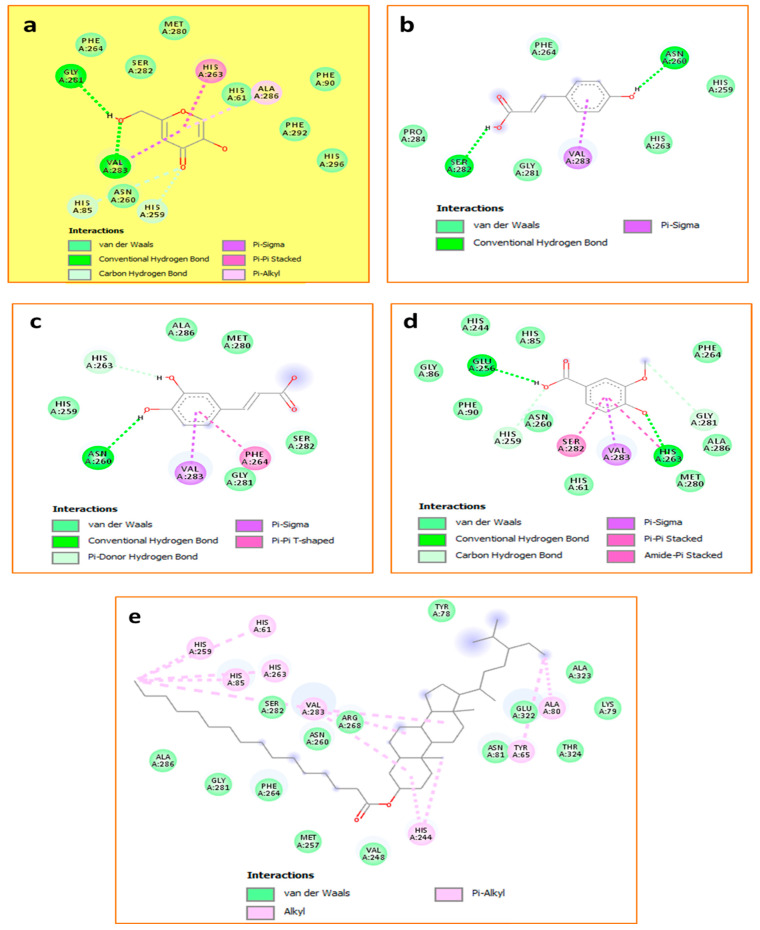
Binding modes of compounds: the reference: kojic acid (**a**), *p*-coumaric acid (**b**), caffeic acid (**c**), vanillic acid (**d**), and *p*-coumaroyl hexose (**e**) within the binding cavity of tyrosinase (PDB: 2y9x).

Against tyrosinase enzyme, and as the tabulated data shows in Table 6, Hhdp-galloyl glucose and vanillic acid were found to be the most effective ligand (−8.1 kcal/mol), showing interesting interactions, such as three H-bonds in addition to a carbon-hydrogen bond, Pi-donor hydrogen bond, and alkyl/Pi-alkyl contacts, as demonstrated in Figure 3 and Figure 4.

The other compounds, *p*-coumaroyl hexose, *p*-coumaric acid, and caffeic acid, although having lower activities than kojic acid, are depicted in Figure 4, which shows that these compounds exhibited significant inhibitory effects through the formation of certain interactions such as Pi-sigma, alkyl/alkyl, and especially hydrogen bonds.

## 3. Discussion

Quinoa leaves, which are extremely rich in phenolic compounds, are of great interest in pharmacology due to their potential antioxidant, anti-Alzheimer, anti-inflammatory, and other health-beneficial properties [2,3,4]. The richness of quinoa leaves in these phenolic compounds makes them a promising research subject for developing new therapeutic agents to improve human health.

In this study, we carried out the extraction of phenolic compounds from quinoa leaves using ethanol and water. Our primary objective was to assess the effect of salt stress on the concentrations of phenolic compounds, with particular emphasis on polyphenols, total flavonoids, and tannins. We also investigated their efficacy in blocking tyrosinases and acetylcholinesterase activities in vitro and in silico. In addition, we sought to determine the antioxidant capacity of these extracts.

The phytochemical study suggested that the aqueous extracts exhibited exceptionally high concentrations of these compounds. This occurrence can be explained by the fact that water is often preferred for extracting more polar compounds, such as polyphenols, tannins, and flavonoids. This property makes it possible to extract compounds with potentially bioactive properties. This solvent has a specific affinity for these compounds, thus facilitating their extraction from plant tissues [20]. The richness of quinoa leaves in metabolites has been confirmed by numerous studies, with the leaves being abundant in bioactive compounds such as polyphenols, flavonoids, saponins, phenolic acids, tannins, alkaloids, and terpenoids. These are present in varying concentrations depending on the environmental conditions and plant maturity [7,8,9]. They are distributed throughout the leaves, making them a valuable source of bioactive compounds.

The results also revealed a significant response of quinoa plants to salt stress, inducing a remarkable increase in phenolic compounds for all solvents used. The progressive increase in saline stress intensity significantly stimulated the total polyphenols, flavonoids, and tannin contents in all studied accessions.

Numerous studies have also confirmed that quinoa plants exposed to salt stress exhibited an increase in the concentrations of secondary metabolites [21,22]. This increase reflects a sophisticated adaptive response to adverse environmental conditions. These metabolites strengthen plants’ resistance to oxidative stress caused by salinity and regulate their growth and development [23,24]. In addition, some phenolic compounds contribute to the regulation of salt stress tolerance by strengthening the cell wall and improving the membrane structure. These defensive and adaptive mechanisms aim to maintain cellular integrity and ensure plant survival under harsh environmental conditions [25]. Increasing levels of phenolic compounds may also play a role in plant defense against opportunistic pathogens that could exploit the plant’s vulnerability in a stressful situation, thereby enhancing the plant’s ability to resist multiple stresses [26,27].

Therefore, the increased response to phenolic compounds may contribute to protection against oxidative damage, ionic regulation, and the prevention of microbial infections. This provides plants with an enhanced ability to cope with multiple stress conditions. This interplay of defense mechanisms enhances plants’ robustness in the face of complex environmental challenges.

Following the quantitative study, the ethanolic and aqueous extracts characterized by significantly high concentrations of phenolic compounds were further evaluated for their efficacy in inhibiting tyrosinase and acetylcholinesterase, as well as their antioxidant activities using ABTS and FRAP tests. The results showed that quinoa leaf extracts had a strong capacity to inhibit the tested enzymes and remarkable antioxidant activities, Similarly, the most attractive efficacy was observed with the aqueous extract. Regarding accessions, Q45 was the most potent extract, exhibiting the lowest IC_50_ values. In fact, under 200 mM, the ethanolic extract achieved IC_50_ values of 1.5 ± 0.05 mg/mL for tyrosinase and 0.53 ± 0.04 mg/mL for acetylcholinesterase, while the aqueous extract recorded values of 1.26 ± 0.14 mg/mL and 0.14 ± 0.002 mg/mL for the same enzymes, respectively.

**Figure 5:**
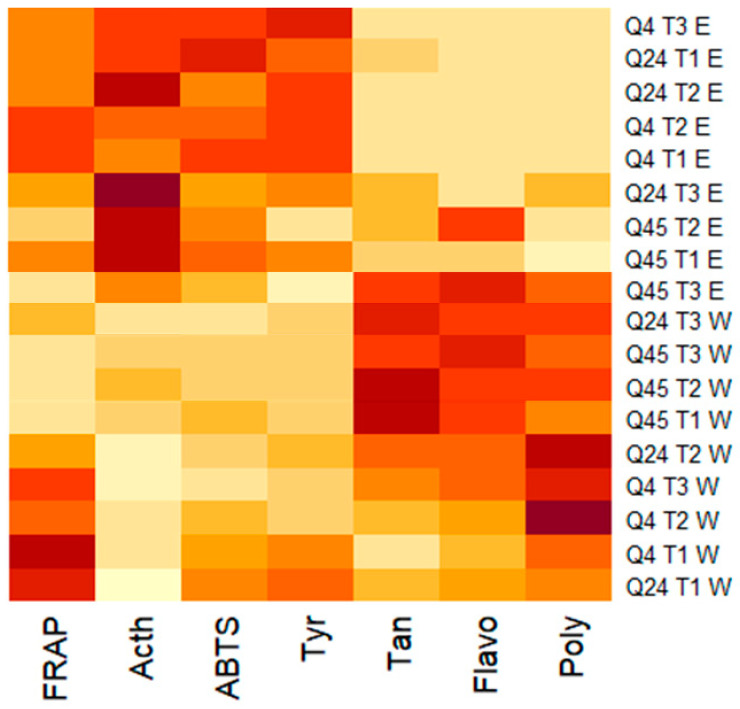
Heat map of the two solvent extracts’ phytochemical parameters and enzymatic activities. E: ethanolic extract; W: aqueous extract; T1: 50 mM; T2: 150 mM; T3: 200 mM; Acth: anti-acetylcholinesterase activity; Tyr: anti-tyrosinase activity; poly: total polyphenol content; Tan; tannin content; Flavo: flavonoid content; ABTS: 2,2′-azinobis (3-ethylbenzothiazoline-6-sulfonic acid); FRAP: Ferric Reducing Antioxidant Power.

The superiority of Q45 over the other two accessions was highlighted by the heat map in Figure 5, which was designed to illustrate the differences and similarities between the quinoa extracts according to the measured parameters.

This graphical representation in the heat map uses a color palette to denote both biological activities and the measured compound concentrations. Light-colored areas indicate low values, while darker shades correspond to high values. The analysis of this map revealed that, regardless of the solvent, Q45 exhibited the darkest colors for polyphenol, flavonoid, and tannin contents while exhibiting the lightest colors for IC_50_ values for biological activities, including tyrosinase and acetylcholinesterase inhibition. These observations, therefore, suggested that Q45 extracts have the highest contents of compounds and a superior ability to block enzymatic activity, which is particularly desirable for therapeutic applications. This trend revealed that in all the studied accessions, an increase in salt stress intensity corresponds to a rise in polyphenol content, as evidenced by the intensification of color. Simultaneously, the IC_50_ values of enzymatic activities decrease, reflected by a reduction in color intensity. Thus, this observation suggests that the elevation in the levels of bioactive compounds was at the expense of enzymatic activity, reinforcing the idea that salt stress stimulates the production of beneficial metabolites while inhibiting enzymatic functions.

**Figure 6:**
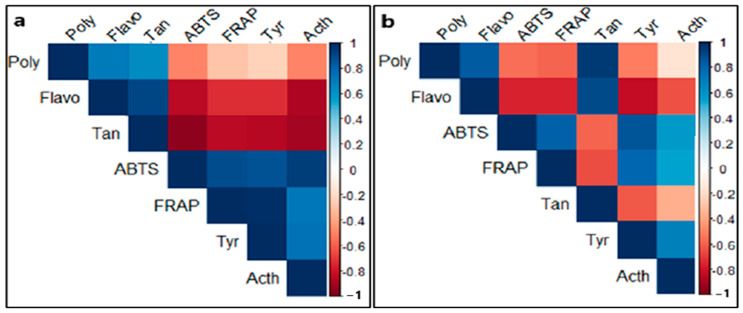
Correlation matrix between the measured parameters in (**a**) ethanolic and (**b**) aqueous extracts. Acth: anti-acetylcholinesterase activity; Tyr: anti-tyrosinase activity; poly: total polyphenol content; Tan: tannin content; Flavo: flavonoid content; ABTS: 2,2′-azinobis (3-ethylbenzothiazoline-6-sulfonic acid); FRAP: Ferric Reducing Antioxidant Power.

This positive relationship between the increase in inhibitory potency of the extracts and the increase in content was also confirmed by the correlation test in Figure 6. The graphs clearly illustrate the existence of a negative correlation between the IC_50_ values of tested enzymes and the contents of the analyzed compounds. As the contents of phytochemical compounds increase, the IC_50_ values decrease. This therefore suggests that the more the extracts are enriched in bioactive compounds, the more they can inhibit enzymatic activity.

Since the aqueous extracts had the highest concentrations of phytochemicals, we selected them to evaluate their major chemical composition by HPLC-DAD-ESI-MS/MS. The results showed the presence of five main compounds. In addition, 200 mM NaCl stimulated caffeic acid and vanillic acid in Q4 by 2.5 and 4.5 times, *p*-coumaric acid in Q24 by 4.5 times, and *p*-coumaroyl hexose and Hhdp-galloyl glucose in Q45 by 7 and 10 times. The stimulating effect of NaCl on caffeic acid and vanillic acid was also observed in the study of Ahmed et al. [28] on *Solanum lycopersicum* L., which reported a 13-fold increase in caffeic acid and a 2-fold increase in vanillic acid under 150 mM NaCl.

To assess the effect of salt stress on the efficacy of the obtained extracts, their activities against tyrosinase and acetylcholinesterase and their antioxidant potential were evaluated.

The results showed that salt stress enhanced the efficacy of the extracts in inhibiting tyrosinase and acetylcholinesterase. This improvement in efficacy underscores the potential of these extracts for applications in the prevention and treatment of pigmentary and neurodegenerative disorders. Numerous studies have confirmed the pharmacological properties of bioactive molecules derived from quinoa leaves, which appear diverse and promising. Indeed, many researchers have suggested that quinoa leaf extracts may have neuroprotective effects, offering the potential to treat neurological disorders such as Alzheimer’s and Parkinson’s diseases [29]. Furthermore, their significant antioxidant activity suggested their potential in preventing diseases related to oxidative stress [30,31]. The phytochemical analysis carried out by HPLC-DAD-ESI-MS/MS identified five major compounds: caffeic acid, vanillic acid, *p*-coumaric acid, *p*-coumaroyl hexose, and Hhdp-galloyl glucose, allowing us to attribute the observed efficacy of the extracts to these compounds. The pharmacological potential of these compounds has been evidenced in numerous studies. Indeed, Jia et al. [32] reported the anti-tyrosinase potential of caffeic acid, and its IC_50_ value was determined to be 37.5 µM. Caruso et al. [33] also showed that vanillic acid demonstrates significant activity in preventing and reducing symptoms associated with Alzheimer’s disease. Similarly, the study of Chang et al. [34] highlights the neuroprotective effects of caffeic acid (CA) against Alzheimer’s disease (AD) in rats. The administration of CA (30 mg/kg/day) for 30 weeks significantly improved learning and memory impairments as demonstrated by the Morris water maze test. The compound enhanced antioxidant defenses by increasing superoxide dismutase and glutathione activity, thereby reducing oxidative stress. In addition, caffeic acid improved synaptic connectivity by upregulating synaptic protein expression. These results suggest that caffeic acid attenuates the progression of Alzheimer’s disease by reducing oxidative stress and improving synaptic function, positioning it as a potential therapeutic agent for Alzheimer’s disease.

Likewise, the antioxidant potential of *p*-coumaric acid and Caffeic acid was proved and assessed using spectrophotometric methods, including ABTS, DPPH, and FRAP assays. These compounds exhibit strong free radical scavenging abilities and a significant capacity to reduce iron and copper ions. Notably, caffeic acid demonstrates superior antioxidant properties, with its activity increasing linearly with concentration. Additionally, caffeic acid is a markedly more potent reducing agent in oxidation processes.

Under salt stress, our results revealed a significant improvement in the efficacy of extracts in inhibiting the target enzymes (tyrosinase and acetylcholinesterase), suggesting an adaptive response of bioactive compounds to stress conditions. This stimulation could be explained by the fact that salt stress can significantly influence the structure, quality, and quantity of secondary metabolites [35], which consequently stimulates the efficacy of these compounds in blocking tyrosinase, acetylcholinesterase, and neutralizing ROS.

This hypothesis was confirmed by the results of HPLC-DAD-ESI-MS/MS analysis, which revealed remarkable changes in the profiles of secondary metabolites under salinity stress conditions. Specifically, some bioactive substances showed an increased concentration, while others decreased or were completely eliminated. This qualitative and quantitative variation has been explained by many authors. Indeed, salt stress can induce changes at the structural level of secondary metabolites. These changes in molecular structure are crucial for their ability to interact specifically with the active sites of these target enzymes. These structural adjustments can influence how metabolites interact with target proteins, thus modulating their inhibitory efficacy. These modifications can include variations in functional groups, molecular bonds, or the spatial configuration of the compounds, which directly impact metabolites’ ability to disrupt enzymatic processes [36].

Furthermore, salt stress can increase the efficiency of the extract by promoting the production of compounds with more pronounced bioactive properties and blocking the synthesis of less active compounds [37]. This hypothesis could explain the remarkable performance of Q45 in these tests despite the apparent simplification of its compounds. Indeed, under 200 mM, only two compounds (*p*-coumaroyl hexose and Hhdp-galloyl glucose) were detected at high concentrations. The observed simplification of the metabolic profile, characterized by a reduction in the total number of compounds, may indeed play a crucial role in improving the extract’s efficacy. When the metabolic profile is simplified, antagonistic interactions between different compounds, which can sometimes reduce overall efficacy, are minimized. Fewer compounds in the extract mean less competition or cross-inhibition between them, allowing key bioactive metabolites to function more efficiently.

The efficacy of secondary metabolites in blocking tyrosinase and acetylcholinesterase may be due to a complex interplay between structural modification, improved metabolite quality, and quantitative increase due to salt stress [38]. Polyphenols exert their inhibitory effects on key enzymes primarily through their ability to interact with the enzyme in several ways. It has been reported that polyphenols interact with the active sites of enzymes, inducing conformational changes that hinder their catalytic activity. By acting as competitive inhibitors, polyphenols compete with standard enzyme substrates to occupy active sites, thereby reducing their effectiveness [39]. Furthermore, further studies have demonstrated that certain polyphenols exert anti-acetylcholinesterase and anti-tyrosinase activity by indirect mechanisms, particularly through anti-inflammatory and antioxidant effects, which contribute to the protection of nerve and skin cells [40,41,42].

This hypothesis was supported by the positive correlation of our results between ABTS and FRAP assays with anti-acetylcholinesterase and anti-tyrosinase activity.

Furthermore, polyphenols may also modulate inflammatory pathways [43,44]. Indeed, chronic inflammation could be associated with many neurological and skin diseases. By attenuating this inflammatory response, polyphenols contribute to the protection of concerned cells by inhibiting the enzymes involved in inflammatory processes, thereby reducing the production of pro-inflammatory mediators such as cytokines and prostaglandins [45,46].

The efficacy of quinoa extract in inhibiting tyrosinase and acetylcholinesterase enzymes was also confirmed by in silico studies, validating our experimental results.

Molecular docking results revealed that hhdp-galloyl glucose was the most bioactive ligand for tyrosinase and acetylcholinesterase, involving various interactions such as hydrogen bonds, Pi-Pi, and alkyl/Pi-Alkyl. The other compounds, *p*-coumaric acid, *p*-coumaroyl hexose, caffeic acid, and vanillic acid, although less active than the standard compounds, showed a significant inhibitory effect through the formation of several interactions, in particular hydrogen bonds and other hydrophobic interactions. These findings highlight the potential of these phytochemicals as potent inhibitors of acetylcholinesterase and tyrosinase, opening up exciting prospects for future therapeutic applications.

## 4. Material and Methods

### 4.1. Material

The study used three quinoa accessions, Q4, Q45, and Q24, whose origin and source were detailed by Slimani et al. [13].

### 4.2. Methods

In this study, quinoa plants were cultivated in a greenhouse environment and subjected to three salinity treatments: 50 mM (control), 150 mM, and 200 mM NaCl. These salinity levels were gradually established through controlled irrigation, considering the watering frequency and the plant’s water requirements, which were recalculated before each irrigation session. Leaves were harvested at the flowering stage and dried at 45 °C in an oven. After drying, they were ground into a fine powder and stored at 4 °C to ensure their suitability for further analysis.

### 4.3. Phenolic Compound Analysis

The protocol for extracting phenolic compounds from quinoa leaves at the flowering stage involves several steps using ethanol and water solvents. Fresh quinoa leaves are harvested and air-dried to retain the phenolic compounds. The dried leaves are then ground into a fine powder, and 100 g of this powdered material is subjected to extraction by maceration. Initially, pure ethanol is used, followed by water, until the extraction is complete and all compounds are exhausted. The resulting extract is filtered through filter paper to remove solid residues and evaporated under reduced pressure using a rotary evaporator at 40–50 °C to concentrate the extract, which is stored at −20 °C for subsequent analysis.

#### 4.3.1. Total Polyphenol Content (TPC) Determination

Quinoa extract (25 µL) was mixed with 125 µL of 0.2 M Folin–Ciocalteu reagent and incubated for 10 min at room temperature. Then, 125 µL of sodium carbonate (Na₂CO₃) solution was added, and the mixture was incubated for 30 [47]. The absorbance was measured at 765 nm, and the total polyphenol content was expressed as milligrams of gallic acid equivalents (mg GAE/100 g dry weight).

#### 4.3.2. Total Flavonoid Content Determination

In brief, 50 µL of quinoa leaf extract was mixed with 50 µL of 2% aluminum chloride solution (dissolved in methanol). The mixture was incubated for 10 min at room temperature, and absorbance was measured at 405 nm [48]. Flavonoid content was expressed as milligrams of catechin equivalents (mg CE/100 g dry weight).

#### 4.3.3. Total Condensed Tannin Content Determination

Condensed tannins were determined using Broadhurst et al.’s (1978) method with slight modifications. A volume of 500 µL of quinoa extract was mixed with 3 mL of 4% vanillin solution (in methanol) and 1.5 mL of concentrated hydrochloric acid. After 15 min of incubation, absorbance was measured at 500 nm [49]. Condensed tannins were expressed as milligrams of catechin equivalents (mg CE/100 g dry weight).

### 4.4. HPLC-DAD-ESI-MS/MS Analysis of the Main Compounds of Water Extract

This analysis was performed as described by [50]. An Agilent 1100 series HPLC system equipped with a diode array detector (DAD), a triple quadrupole mass spectrometer type Micromass Autospec Ultima Pt (Kelso, UK), and an ESI ion source operating in negative mode was used for the identification of phenolic compounds. The mobile phase consisted of A (0.1% acetic acid) and B (acetonitrile) with a flow rate of 0.25 mL/min. Separation was achieved using a Superspher^®^100 column (12.5 cm × 2 mm i.d, 4 µm, Agilent Technologies, Rising Sun, MD, USA) at 45 °C with a multistep linear gradient elution program in which phase B changed from 0 to 2% in 5 min, from 2 to 88% in 75 min, and from 88 to 2% in 90 min. The Uv-Vis spectra were recorded from 200 to 800 nm, and ions in the *m*/*z* range of 100–1000 were detected using a scan time of 1 s. The ESI source was conducted under the following operating conditions: capillary voltage, 3.2 kV; cone voltage, 115 V; probe temperature, 350 °C; and ion source temperature, 110 °C. Data acquisition was achieved with a Masslynx software version 4.0. The tentative identification of phenolics was carried out by considering their UV and mass spectra and comparing their retention time and fragmentation pattern with those of authentic standards when available and/or literature data.

### 4.5. Enzyme Activity Assays

#### 4.5.1. In Vitro Study

- Acetylcholinesterase inhibitory activity: A reaction mixture was prepared with 20 µL AChE solution (0.5 units/mL), 20 µL extract, 40 µL 0.1 mM phosphate buffer (pH 8.0), and 20 µL 0.2 M DTNB. This mixture was incubated for 15 min at 25 °C. The reaction was then initiated by adding 10 µL of 0.2 M acetylthiocholine iodide, allowing monitoring of the enzymatic activity by the formation of the yellow 5-thio-2-nitrobenzoate anion [51]. Absorbance was measured at 412 nm using galanthamine as a reference.
- Tyrosinase inhibitory activity: For this assay, 40 µL of 1.5 mM L-tyrosine solution was mixed with 140 µL of 0.2 M phosphate buffer (pH 6.8) and incubated at 25 °C for 10 min. Then, 100 µL of extracts and 20 µL of mushroom tyrosinase (1500 units/mL in 0.2 M phosphate buffer, pH 6.8) were added. The mixture was mixed thoroughly and incubated at 37 °C for 15 min [52]. Optical density was recorded at 492 nm using kojic acid as a reference.
- Antioxidant activity tests:
  2.2′-azino-bis (3-ethylbenzothiazoline-6-sulphonic acid) (ABTS +•) assay: A 7 mM ABTS solution was mixed with 2.45 mM potassium persulfate (1:1. *v*/*v*). After 16 h at room temperature, the solution was then diluted with ethanol to give an absorbance of 0.70 ± 0.02 at 734 nm. For the assay, 50 µL of the extract was added to 950 µL of ABTS solution and incubated for 15 min at room temperature [48]. Absorbance was recorded at 734 nm.
  Ferric reducing ability of plasma (FRAP assay): The FRAP reagent was prepared by mixing 5 mL of 10 mM TPTZ (dissolved in 40 mM HCl) with 5 mL of 20 mM ferric chloride and 50 mL of 300 mM acetate buffer (pH 3.6) and then heating to 37 °C. A 150 µL extract was added to a 2,580 µL FRAP reagent, and the absorbance was measured at 593 nm after 15 min [53].

#### 4.5.2. In Silico Study: Molecular Docking Procedure

Molecular docking simulations were performed using Auto Dock 4.2 software [54] to investigate the binding interactions between the selected identified bioactive phytocompounds and various enzymes. The protocol employed was identical to that described previously by Aouadi et al. [55]. The three-dimensional structures of tyrosinase (PDB: 2y9x) and acetylcholinesterase (PDB: 4ey7) were downloaded from the RCSB Protein Data Bank.

### 4.6. Statistical Analysis

All data were subjected to a one-way analysis of variance using SPSS 26.0 software, and differences between means were compared by Duncan’s test (*p* < 0.05).

Correlation analyses and heat maps between the different parameters analyzed were performed using R software (version 4.3.0; RStudio 2024.04.1+748).

## 5. Conclusions

In conclusion, this study demonstrates quinoa’s ability to maintain and even enhance its phenolic content under salinity stress. This was characterized by an increase in the concentrations of polyphenols, flavonoids, and tannins. Among the accessions, Q45 stands out for its richness in phenolic compounds, underlining the adaptive response of this accession. The enrichment of the phytochemical profile of the quinoa leaf underlines its potential as a reliable source of therapeutic compounds, even under adverse environmental conditions.

It was also found that salinity increased the concentration of these compounds and enhanced their biological activity. Specifically, leaves exposed to higher salt concentrations exhibited a greater ability to inhibit enzymes such as tyrosinase and acetylcholinesterase along with strong antioxidant activity. Additionally, accession Q45 demonstrates the highest inhibitory potential.

This study therefore reinforces the interest in using plant resources for the development of new medical therapies by demonstrating that even under environmental challenges, plants such as quinoa can not only survive but also produce bioactive compounds with increased efficacy, reduced toxicity to the environment, and fewer side effects for patients. This could pave the way for a new perspective in pharmaceutical research where nature, in all its resilience and complexity, becomes a source of inspiration and solutions to the health challenges of the 21st century, combining environmental sustainability and therapeutic efficacy.

In light of these results, it would be relevant to conduct in vivo research to confirm the efficacy of the bioactive compounds extracted from quinoa leaves under real physiological conditions, verifying their bioavailability, metabolism, and long-term pharmacological effects.

## Figures and Tables

**Table 1:** Contents in polyphenols, flavonoids, and tannins in quinoa leaf extracts under salt stress.

		Ethanol			Water		
	Treatments (mM of NaCl)	Q4	Q24	Q45	Q4	Q24	Q45
Flavonoid content (mg CAE/100 g DW)	50	250.4 ± 33.3 cB	261.5 ± 25.5 cB	322.7 ± 42 cA	406.2 ± 42 bA	439.6 ± 58 cA	467.44 ± 16.6 cA
	150	300.5 ± 44.1 bC	361.7 ± 25.5 bB	578.7 ± 34.7 bA	450.7 ± 33.6 bC	589.8 ± 53.6 bB	751.25 ± 16.6 bA
	200	367.2 ± 16.7 aC	478.5 ± 25.5 aB	751.2 ± 44.1 aA	617.6 ± 16.7 aC	784.6 ± 44.16 aC	1090.7 ± 53.6 aA
Total polyphenol contents (mg AGE/100 g DW)	50	1295.9 ± 115.5 cB	1924.2 ± 179 cA	1453.7 ± 69.4 cB	3141.8 ± 180 cA	2670.4 ± 245 bB	2513.3 ± 179 bB
	150	1806.4 ± 136 bB	2316.6 ± 180 bA	2356.1 ± 116 bA	4005.6 ± 310 aA	4123.4 ± 424 aA	4005.6 ± 471 aA
	200	2316.9 ± 169 aC	3141.6 ± 180 aB	3377.7 ± 245 aA	3691.4 ± 173 bB	4202.8 ± 241 aA	4398.5 ± 135 aA
Tannin content (mg CAE/100 g DW)	50	55.6 ± 8.9 bC	116.8 ± 16.6 bA	77.9 ± 34.7 cB	94.6 ± 25.5 cC	133 ± 15.9 cB	183 ± 16.1 bA
	150	100.1 ± 16.6 aB	105.7 ± 9.8 bB	144.6 ± 8.9 bA	133,5 ± 16.7 bC	211.4 ± 7.8 bB	333.8 ± 33.3 aA
	200	105.7 ± 9 aC	183.6 ± 28.9 aB	230.5 ± 5.7 aA	178 ± 25.5 aC	278.2 ± 19.2 aB	335.96 ± 9.8 aA

Mean comparisons based on Duncan’s test were calculated between accessions (uppercase letters) and between treatments (lowercase letters) (a, b, c; A, B, C). According to Duncan’s test, means followed by the same letter are not significantly different at *p* < 0.05.

**Table 2:** Profile of the main compounds identified by HPLC-DAD-ESI-MS/MS in quinoa water extracts.

					Percentages (%)					
					Q4		Q24		Q45	
Major Compound Identified	RT	Uv	[M-H]-	Main Fragments	50 mM	200 mM	50 mM	200 mM	50 mM	200 mM
***p*** **-Coumaric acid**	2.71	275	163	118	10.56 ± 0.4 aB	0.22 ± 0.02 bB	4.37 ± 0.22 bC	19.4 ± 0.6 aA	12.2 ± 0.26 aA	ND aC
**Caffeic acid**	3.21	322	179	135	11.45 ± 0.5 bC	28.51 ± 0.25 aB	40.9 ± 0.13 aA	34.16 ± 0.29 bA	30.26 ± 0.25 aB	ND aC
**Vanillic acid**	4.03	264	167	152	4.16 ± 0.05 bC	18.11 ± 0.45 aA	15.83 ± 0.05 aA	12.13 ± 0.05 bB	8.5 ± 0.01 aB	ND aC
***p*** **-coumaroyl hexose**	32.08	275	519	325. 163	1.91 ± 0.09 aB	0.38 ± 0.015 bC	1.4 ± 0.015 bC	5.99 ± 0.05 aB	3.48 ± 0.02 bA	24.16 ± 0.03 aA
**Hhdp-galloyl glucose**	33.65	254	633	463. 301	1.14 ± 0.02 bA	9.51 ± 0.04 aA	0.17 ± 0.01 bC	0.51 ± 0.01 aC	0.42 ± 0.02 bB	4.3 ± 0.15 aB

Mean comparisons based on Duncan’s test were calculated between accessions (uppercase letters) and between treatments (lowercase letters) (a, b; A, B, C). According to Duncan’s test, means followed by the same letter are not significantly different at *p* < 0.05.

**Table 3:** IC_50_ (mg/mL) of ethanol and water extracts of quinoa leaves for ABTS and FRAP assays.

	Solvants		Accessions			
		Treatments (mM)	Q4	Q24	Q45	
ABTS	Ethanol	50	7.46 ± 0.56 aA	7.76 ± 0.28 aA	4.83 ± 0.32 aB	BHT = 0.01741 ± 0.00055 ccc ccc
		150	6.9 ± 0.11 bA	5.5 ± 0.88 bB	4.73 ± 0.72 abC	
		200	7.1 ± 0.11 abA	5.1 ± 0.4 bB	4.5 ± 0.4 bB	
	Water	50	4.3 ± 0.89 aA	4.26 ± 0.28 aA	3.23 ± 0.42 aB	
		150	4.03 ± 0.28 aA	3.66 ± 0.34 bB	2.13 ± 0.23 bC	
		200	3.4 ± 0.11 bA	3.23 ± 0.23 bB	2.16 ± 0.17 bC	
FRAP	Ethanol	50	1.02 ± 0.04 abA	0.82 ± 0.12 aB	0.67 ± 0.02 aC	BHT = 0.023 ± 0.001ccc cdd
		150	1.1 ± 0.11 aA	0.81 ± 0.04 aB	0.65 ± 0.04 aC	
		200	0.86 ± 0.23 bA	0.77 ± 0.02 bA	0.57 ± 0.05 bB	
	Water	50	0.84 ± 0.05 aA	0.76 ± 0.01 aB	0.49 ± 0.04 aC	
		150	0.77 ± 0.02 bA	0.7 ± 0.01 bB	0.37 ± 0.03 bC	
		200	0.86 ± 0.23 aA	0.63 ± 0.02 cB	0.28 ± 0.02 cC	

Mean comparisons based on Duncan’s test were calculated between accessions (uppercase letters) and between treatments (lowercase letters) (a, b, c, d; A, B, C). According to Duncan’s test, means followed by the same letter are not significantly different at *p* < 0.05. Colors are used to compare the antioxidant activity found between treatments for each accession with ethanol or water extract with the BHT.

**Table 4:** IC_50_ (mg/mL) of ethanol and water extracts of quinoa leaves for tyrosinase and acetylcholinesterase activities.

			Accessions			Standard
Enzyme	Solvants	Treatments (mM)	Q4	Q24	Q45	
Tyrosinase	Ethanol	50	4.06 ± 0.37 aA	3.3 ± 0.2 bA	2.23 ± 0.07 cA	Kojic acid: 0.178 ± 0.039 CCD CDC
		150	3.83 ± 0.51 aB	3.2 ± 0.2 bA	1.88 ± 0.08 cB	
		200	3.96 ± 0.15 aAB	2.7 ± 0.2 bB	1.5 ± 0.05 cC	
	Water	50	2.26 ± 0.05 aA	2.2 ± 0.05 aA	1.5 ± 0.05 bA	
		150	1.9 ± 0.1 aB	2 ± 0.08 aB	1.28 ± 0.16 bB	
		200	1.82 ± 0.06 aB	1.76 ± 0.2 aC	1.26 ± 0.14 bB	
Acetylcholinesterase	Ethanol	50	0.68 ± 0.07 bC	0.88 ± 0.09 aA	0.66 ± 0.07 bA	Galanthamine: 0.013 ± 0.002 DCD CDD
		150	0.83 ± 0.1 bB	0.9 ± 0.05 aA	0.61 ± 0.036 cB	
		200	0.9 ± 0.05 aA	0.82 ± 0.01 bB	0.53 ± 0.04 cC	
	Water	50	0.21 ± 0.02 aA	0.22 ± 0.02 aA	0.18 ± 0.01 bA	
		150	0.22 ± 0.02 aA	0.19 ± 0.01 bB	0.15 ± 0.005 cB	
		200	0.17 ± 0.01 aB	0.16 ± 0.005 aC	0.14 ± 0.002 bC	

Mean comparisons based on Duncan’s test were calculated between accessions (lowercase letters) and between treatments (uppercase letters) (a, b, c; A, B, C, D). According to Duncan’s test, means followed by the same letter are not significantly different at *p* < 0.05. Colors are used to compare the enzyme activity found between treatments in each accession for ethanol or water extract with the Standard.

**Table 5:** Binding energy of the docked compounds in the binding cavity of acetylcholinesterase (PDB: 4ey7).

Compound	Binding Energy (kcal/mol)
*p*-coumaric acid	−6.8
Caffeic acid	−7.1
Vanillic acid	−6.2
*p*-coumaroyl hexose	−8.9
Hhdp-galloyl glucose	−11.5
Galantamine	−8.6

**Table 6:** The binding energy of the docked compounds in the binding cavity of tyrosinase (PDB: 2y9x).

Compound	Binding Energy (kcal/mol)
***p*-coumaric acid**	−4.7
**Caffeic acid**	−4.9
**Vanillic acid**	−5.9
***p*-coumaroyl hexose**	−5.4
**Hhdp-galloyl glucose**	−8.1
**kojic acid**	−5.5

## Data Availability

The data supporting the findings of this study are available from the corresponding author upon reasonable request.
